# IJS: An Intelligent Junction Selection Based Routing Protocol for VANET to Support ITS Services

**DOI:** 10.1155/2014/653131

**Published:** 2014-10-29

**Authors:** Sourav Kumar Bhoi, Pabitra Mohan Khilar

**Affiliations:** Department of Computer Science and Engineering, National Institute of Technology, Rourkela 769008, India

## Abstract

Selecting junctions intelligently for data transmission provides better intelligent transportation system (ITS) services. The main problem in vehicular communication is high disturbances of link connectivity due to mobility and less density of vehicles. If link conditions are predicted earlier, then there is a less chance of performance degradation. In this paper, an intelligent junction selection based routing protocol (IJS) is proposed to transmit the data in a quickest path, in which the vehicles are mostly connected and have less link connectivity problem. In this protocol, a helping vehicle is set at every junction to control the communication by predicting link failures or network gaps in a route. Helping vehicle at the junction produces a score for every neighboring junction to forward the data to the destination by considering the current traffic information and selects that junction which has minimum score. IJS protocol is implemented and compared with GyTAR, A-STAR, and GSR routing protocols. Simulation results show that IJS performs better in terms of average end-to-end delay, network gap encounter, and number of hops.

## 1. Introduction

Vehicular ad hoc network (VANET) is an advance wireless communication network to provide safety to the passengers and drivers [1–5]. Due to the high population of vehicles in the city areas, the level of the accident increases. This reduces the safety level for passengers and drivers in the city environment. So, there should be a communication between the vehicles, so that a vehicle which encounters an accident can send a warning message to the fellow vehicles to prevent the vehicles from an accident. VANET provides many types of applications to the users by using ITS services [6, 7]. Vehicles provide services [8] like P2P communication, media downloading, safety systems, vehicular cloud computing, e-marketing, and so forth. VANET uses many types of communication like vehicle-to-vehicle communication (V2V), vehicle-to-infrastructure/infrastructure-to-vehicle communication (V2I/I2V), and infrastructure-to-infrastructure (I2I) communication. The architecture of VANET consists of road side units (RSUs) which acts as the infrastructure to communicate with the vehicles and central authority (CA). Many projects are developed and implemented in USA and Japan and the European nations like WAVE, IVI, VSC, ASV-2, C2C-CC, Fleetnet, NoW, iTETRIS, and so forth to provide ITS services to the users [1, 3]. The main objective of these projects is to provide safety applications to the passengers and drivers.

VANET mainly suffers from link connectivity problem due to high mobility and less density of vehicles in an area [9]. Drivers are of many types in the city areas like aggressive drivers, slow drivers, and moderate drivers [5, 10]. Due to the speed of these drivers there is high mobility which leads to network gaps. Network gap is a zone where a vehicle is unable to communicate with its forward positioning vehicles. This is called link connectivity problem where no link is available between the vehicles to communicate. As topology frequently changes, topology based routing protocols are difficult to implement on VANET environment. So, position based routing protocols are used to send the data to the vehicles by knowing their location.

The main objective of this paper is to select the most connected route which has less link connectivity problems or network gaps. This enhances the performance of the system by reducing the delay. The main contribution in this paper is as follows.

(1) An IJS routing protocol is proposed to transmit the data quickly to the destination *D* in the mostly connected path which has less number of network gaps. An HV is set at every junction to predict the link failures (network gaps) between the vehicles in a route (between two neighboring junctions).
(2) HV calculates the score, Score, for every neighboring junction by predicting the delay between the two junctions and predicting the link connectivity status between the vehicles in a logical route Route_reference_ (logical route from one junction to another). HV selects the junction which has a minimum Score and forwards the data in that direction. This process continues until last junction *J*_last_ (junction nearer to *D*) is reached and then data is sent in the route of *D* through the vehicles.

The paper is organized as follows.  Section 2 presents the related works which discuss the standard routing protocols proposed for forwarding the data to destination.  Section 3 presents the preliminary part in which three basic models are discussed which are the key elements for IJS routing protocol.  Section 4 presents the IJS routing protocol where system model and functionality of the model are described.  Section 5 presents the analysis of the proposed IJS routing protocol.  Section 6 presents the simulation part where the protocols are implemented and compared to identify the performance. At last, we concluded in Section 7.

## 2. Related Works

Many works are proposed to provide efficient routing protocols for VANET to send the data quickly from the source to the destination [3, 11, 12]. We are motivated by an efficient junction routing protocol known as GyTAR proposed by Jerbi et al. [13] to send the data efficiently through the junctions. A score is calculated for every neighboring junction by knowing the current traffic conditions. This protocol performs better because the vehicles near the junctions are aware of the traffic conditions. Cell data packet (CDP) is used mainly to send the density information to the junctions. But there may be many network gaps generated between the junctions by which the CDP information is not updated at the current time. This may generate a false score calculation. The congestion in the network is more due to high CDP packet exchange.

A-STAR [14] is a junction routing protocol in which the data packet is transmitted in a predefined path which consists of buses and vehicles. These junctions have higher weightage than the normal junctions. So, the vehicles always prefer to send the data through the densified regions. But by using these routes there may be delay in reaching the destination because these types of routes exist in some areas. There may be less density in these areas which leads to higher delay.

GSR routing protocol [14] is a position based routing protocol, which sends the data in the shortest path route to *D*. This suffers from link connectivity problem due to lack of information about the traffic conditions ahead. This also increases the delay.

GPSR [15] is a position based routing protocol, which forwards the data by selecting the vehicle which is nearer to the destination. To overcome the local optimum problem, it works in perimeter mode. Due to the lack of information about the current traffic conditions, it sometimes goes to perimeter mode. This degrades the performance of the network.

GPCR [16] is a routing protocol, which chooses a coordinator node to forward the data to the destination. After forwarding the data through the street, vehicles choose a coordinator node at the junction. When the density of the vehicles in a region is low, GPCR suffers from the network gap problem. It does not use current traffic information to choose the best path to the destination.

Chen et al. [17] proposed a diagonal-intersection-based routing protocol (DIR) in which the source forwards the data through the diagonal junctions. The path to the diagonal junction is selected by finding a delay and the path with less delay is selected as the path to reach the diagonal junction. This process continues until destination is reached. This autoadjustability enhances the performance of the system.

Bernsen and Manivannan [18] proposed a reliable intervehicular routing (RIVER) protocol by considering the current traffic information. It assigns a reliability rating to the street edges and forwards the data according to the ratings. It shares the reliability information with other vehicles to make other vehicles aware of the current traffic conditions. This increases the performance of the system by selecting the best possible routes to the destination.

Eiza and Ni [19] proposed a graph based reliable routing protocol in which evolving graph theory is used to forward the data to the destination in a highway scenario. This protocol is mainly proposed to provide QoS in routing. It provides better average end-to-end delay, packet delivery ratio, and lower link failure rate.

Saleet et al. [20] proposed an intersection-based geographical routing protocol (IGRP) to send the data effectively through the junction to reach the internet gateway. The junctions are selected on the basis of delay, QoS, bandwidth usage, connectivity, and error rate.

Bilal et al. [21] proposed an enhanced-GyTAR routing protocol to send the data through the intelligent junctions by finding a score. But this protocol only considers the density and speed of the vehicles. It does not know about the connectivity of the vehicles. CDP packet is used for connectivity information but due to network gaps the information is not updated which leads to false score calculation.

Taleb et al. [22] proposed a stable routing protocol where the vehicles are grouped according to their velocity vectors. This method enhances the connectivity between the vehicles. This leads to less link breakage problems. This model reduces the traffic by reducing the broadcast storm in the network. The main idea of this method is to send the well-defined packets.

In summary, if the traffic conditions are predicted then there is a less chance of performance degradation. Many protocols predict the route by knowing the density of the vehicles. If in a road (connecting two junctions) there is high density then this does not signify that the vehicles are connected. IJS routing protocol predicts the link connectivity status LCS between the two vehicles in a route in high density and low density. This LCS is used to calculate the score of a neighboring junction. It predicts the delay to send the data from one junction to the other and this delay is used to calculate the score. The junction with the minimum Score is selected as the optimal junction through which the data is forwarded to *D*. To calculate the score, IJS uses the three basic models discussed in the next section. The notations used in the paper are shown in Notations section.

## 3. Preliminaries

### 3.1. Expected Data Transmission Delay Model (EDTDM)

This model shows the expected time to send a message of length *L* in V2V communication and V2I/I2V communication [23]. The expected time *t*_expected(*V*_1_,*V*_2_)_ to send a message of length *L* bits from one vehicle *V*_1_ to the other vehicle *V*_2_ at a data transmission rate of *r*_(*V*_1_,*V*_2_)_ is

(1) $$\begin{array}{c} t_{\text{expected}(V_{1},V_{2})}=\frac{L}{r_{\left(V_{1},V_{2}\right)}}+\frac{d_{\left(V_{1},V_{2}\right)}}{P_{\text{speed}}}+t_{\text{other}}. \end{array}$$

*L*/*r*_(*V*_1_,*V*_2_)_ denotes the transmission delay and *d*_(*V*_1_,*V*_2_)_/*P*_speed_ denotes the propagation delay, where *d*_(*V*_1_,*V*_2_)_ denotes the distance between the two vehicles $\left(\sqrt{{\left(x_{1}-x_{2}\right)}^{2}+{\left(y_{1}-y_{2}\right)}^{2}}\right)$, where (*x*_1_, *y*_1_) and (*x*_2_, *y*_2_) denote the position coordinates of the vehicles, *P*_speed_ denotes the propagation speed, and *t*_other_ denotes the other delays representing the queuing delay and processing delay. The expected time *t*_expected(*V*,RSU)_ to send a message of length *L* bits from a vehicle *V* to RSU (V2I) at a data transmission rate of *r*_uplink(*V*,RSU)_ (uplink communication) is

(2) $$\begin{array}{c} t_{\text{expected}(V,\text{RSU})}=\frac{L}{r_{\text{uplink}(V,\text{RSU})}}+\frac{d_{\left(V,\text{RSU}\right)}}{P_{\text{speed}}}+t_{\text{other}}. \end{array}$$

For downlink transmission from RSU to a vehicle *V* at rate *r*_downlink(RSU,*V*)_, *t*_expected(RSU,*V*)_ is calculated to be

(3) $$\begin{array}{c} t_{\text{expected}(\text{RSU},V)}=\frac{L}{r_{\text{downlink}(\text{RSU},V)}}+\frac{d_{\left(\text{RSU},V\right)}}{P_{\text{speed}}}+t_{\text{other}}. \end{array}$$

The expected time *t*_expected(RSU_1_,RSU_2_)_ to send a message of length *L* bits from one RSU to another (I2I) at a data transmission rate of *r*_(RSU_1_,RSU_2_)_ is

(4) $$\begin{array}{c} t_{\text{expected}(\text{RS}{\text{U}}_{1},\text{RS}{\text{U}}_{2})}=\frac{L}{r_{\left(\text{RS}{\text{U}}_{1},\text{RS}{\text{U}}_{2}\right)}}+\frac{d_{\left(\text{RS}{\text{U}}_{1},\text{RS}{\text{U}}_{2}\right)}}{P_{\text{speed}}}+t_{\text{other}}. \end{array}$$

V2I, I2V/V2I, and I2I communication enhances the network performance by preventing the network from network gap problem. If a vehicle *V*_1_ is not in a communication range of another vehicle *V*_2_, then network gap is created and *V*_2_ can transmit the data to RSU (to prevent network gap problem). Then, RSU transfers the data to another vehicle or RSU. As RSU has a higher communication range than a vehicle, it sends the data to a vehicle which is nearer to *D* (*D* is the neighboring junction). This reduces the delay to send the data from one junction to the other.

Let the vehicles which are selected as the next hop vehicles between the two junctions to transmit the data be *V* = (*V*_1_, *V*_2_,…, *V*_*n*_), where *n* is the number of next hop vehicles. Then, the expected time delay to send the data from one junction *J*_current_ to another junction *J*_neighbor_ using V2V communication is

(5) texpected(Jcurrent,Jneighbor)=Lr(V1,V2)+d(V1,V2)Pspeed+tother+Lr(V2,V3)  +d(V2,V3)Pspeed+tother+⋯+Lr(Vn−1,Vn)+d(Vn−1,Vn)Pspeed+tother.

As data transmission through a route has different data rates (V2V, V2I, I2V, and I2I), the total expected time delay to send the data packet from one junction *J*_current_ to another junction *J*_neighbor_ using V2V, V2I, I2V, and I2I communication is

(6) texpected(Jcurrent,Jneighbor)=(Lr(V1,V2)+d(V1,V2)Pspeed+tother+⋯   +Lr(Vn−1,Vn)+d(Vn−1,Vn)Pspeed+totherd(Vn−1,Vn)Pspeed) +(Lruplink(V1,RSU1)+d(V1,RSU1)Pspeed+tother+⋯   +Lruplink(Vm,RSUm)+d(Vm,RSUm)Pspeed+tother) +(Lrdownlink(RSU1,V1)+d(RSU1,V1)Pspeed+tother+⋯   +Lrdownlink(RSUi,Vi)+d(RSUi,Vi)Pspeed+tother) +(Lr(RSU1,RSU2)+d(RSU1,RSU2)Pspeed+tother+⋯   +Lr(RSUk−1,RSUk)+d(RSUk−1,RSUk)Pspeed+tother)=∑n=1ntexpected(Vn−1,Vn)+∑m=1mtexpected(Vm,RSUm) +∑i=1itexpected(RSUi,Vi)+∑k=1ktexpected(RSUk−1,RSUk),

where *m* is the total number of data forwarding operations from vehicle *V* to RSU, *i* is the total number of data forwarding operations from RSU to vehicle *V*, and *k* is the total number of RSUs. In this model, only transmission delay and propagation delay are considered as the main performance parameters. We have used this model for expecting the data transmission delay to calculate the score.

### 3.2. Link Connectivity Model (LCM)

The link connectivity model (LCM) describes whether a link is available between the vehicles or not. Let at time *t* the link start and continue up to *t*_1_ time. So, the link availability time between the two vehicles *t*_available_ is denoted by

(7) $$\begin{array}{c} t_{\text{available}}=t+t_{1}. \end{array}$$

The velocity of the vehicles is used to calculate the link connectivity time between the two vehicles. The IJS protocol only considers the vehicles which are moving in the same direction. This means that the data is transmitted to the vehicle which is in the forward position. Let the positions of the vehicles *V*_1_ and *V*_2_ at time *t* be (*x*_1_, *y*_1_) and (*x*_2_, *y*_2_), respectively. Let *V*_1_ and *V*_2_ move at a speed of *v*_1_ and *v*_2_, respectively, and *R* denotes the communication range of a vehicle which is the same for all the vehicles. There are two cases to calculate the connectivity time between the vehicles.

*Case 1*. *V*_1_ is ahead of *V*_2_ and *V*_1_ moves faster than *V*_2_:

(8) $$\begin{array}{c} t_{\text{available}}=t+\frac{R-\sqrt{{\left(x_{1}-x_{2}\right)}^{2}+{\left(y_{1}-y_{2}\right)}^{2}}}{\left|v_{1}-v_{2}\right|}. \end{array}$$

*Case 2*. *V*_1_ is ahead of *V*_2_ and *V*_2_ moves faster than *V*_1_:

(9) $$\begin{array}{c} t_{\text{available}}=t+\frac{\sqrt{{\left(x_{1}-x_{2}\right)}^{2}+{\left(y_{1}-y_{2}\right)}^{2}}}{\left|v_{2}-v_{1}\right|}. \end{array}$$

From (8) and (9), if *t*_available_ > 0, link *l* exists, else the link is disconnected. According to IJS, LCS is set according to the conditions in (10). This model is used to predict the LCS values of the links in a route and used to calculate the score:

(10) $$\begin{array}{c} \text{LCS}=\{\begin{array}{cc} 1, & \text{if}\;\;t_{\text{available}}>0\\ 0, & \text{if}\;\;t_{\text{available}}\leq 0. \end{array} \end{array}$$

The connectivity probability *Pr*⁡ of a road is calculated from the vehicles between the junctions [13]. This connectivity signifies how much the vehicles are connected to each other to form a path from one junction to the other according to their radio range. The distance *d*_(*V*_1_,*V*_2_)_ between the vehicles is expressed in terms of exponential distribution and it is shown as follows:

(11) $$\begin{array}{c} f\left(d_{\left(V_{1},V_{2}\right)}\right)=\lambda e^{-\lambda d_{\left(V_{1},V_{2}\right)}}, \end{array}$$

where *λ* denotes the density of the vehicles per kilometer. The probability that a vehicle is in the range *R* of another vehicle is

(12) $$\begin{array}{c} \mathrm{Pr}=\overset{R}{\underset{0}{\int }}f\left(d_{\left(V_{1},V_{2}\right)}\right)=1-e^{-\lambda R}. \end{array}$$

So, (13) shows the probability of a link *l* between the two vehicles when they are connected. The probability Pr′ that the vehicles are not connected is shown as follows:

(13) $$\begin{array}{c} \text{P}{\text{r}}^{\prime }=e^{-\lambda R}. \end{array}$$

If the vehicles on a road are connected consecutively to form a path from one junction to the other, then the probability of connectivity of the road is

(14) $$\begin{array}{c} {\mathrm{Pr}}_{\text{road}}={\left[1-e^{-\lambda R}\right]}^{n}, \end{array}$$

where *n* is the number of vehicles. If a road segment consists of *n* vehicles which form a path by connection consecutively then there are *n* − 1 number of links. If *p* number of links fails, then the probability of connectivity of the road is

(15) $$\begin{array}{c} {\mathrm{Pr}}_{\text{road}}={\left[1-e^{-\lambda R}\right]}^{n-1-p}\cdot {\left[e^{-\lambda R}\right]}^{p}. \end{array}$$

### 3.3. Vehicle Population Model (VPM)

**Figure 1:**
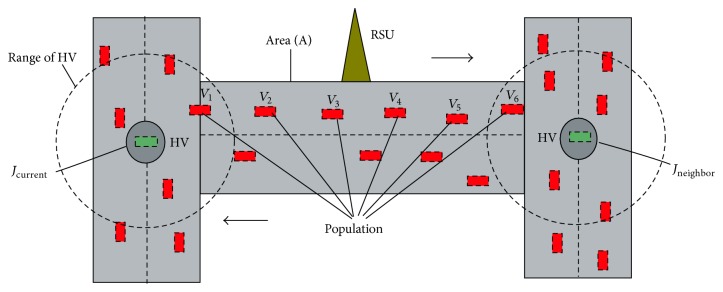
Population between the two junctions.

This section shows the population *U* of the vehicles between any two junctions and their positions. From Figure 1, let the population of the vehicles in an area *A* at an interval *t*_*U*_ be given by *U* = (*V*_1_, *V*_2_, *V*_3_, *V*_4_, *V*_5_, *V*_6_). In this model, a fixed vehicle HV is set at every junction which records the traffic information using beaconing service, where every vehicle beacons to share its location, speed, direction, and identity. Let the speed of the vehicles vary from *v*_1_ to *v*_2_ and the vehicles can attain any speed between them (government is limiting the speed to control the accidents). At any time *t*_*U*_, the population of the vehicles in area *A* is calculated by predicting the positions of the vehicles between the junctions. HV knows the positions of the vehicles which are in the range. So, HV predicts the positions of the vehicles (out of range vehicles) by calculating the distance covered by a vehicle after the last beacon. Last beacon signifies the last information broadcasted by a vehicle to its neighboring vehicles after which the vehicle becomes out of range. From this information, HV knows the speed of the vehicle at the time *t*_last-beacon_. The position of the vehicle is predicted by

(16) $$\begin{array}{c} d_{\text{covered}}=\left(t_{U}-t_{\text{last-beacon}}\right)v_{\text{last-beacon}}, \end{array}$$

where *d*_covered_ is the distance covered by a vehicle after last beacon and *v*_last-beacon_ signifies the speed of the vehicle presented in the last beacon. By finding the *d*_covered_ by all the vehicles between the two junctions, population *U* can be calculated. But for how many vehicles HV calculates the *d*_covered_ and finds *U*. This can be calculated by finding a time *t*_cross_, which signifies the time taken to cross another junction by a vehicle. If in between the two junctions the vehicles move at a speed limit of *v*_1_ to *v*_2_, then in the worst case *t*_cross_ is calculated to be

(17) $$\begin{array}{c} t_{\text{cross}}=\frac{P_{\text{length}}}{v_{1}}, \end{array}$$

where *P*_length_ is the path length from one junction to another and *v*_1_ is the lowest bound speed. So, *d*_covered_ is calculated for the vehicles which crosses the junction between *t*_*U*_ − *t*_cross_ and *t*_*U*_. IJS considered only those vehicles whose positions are between the junctions. This model is used to calculate the positions of the vehicles moving in a direction and used to find a Route_reference_ from one HV to the other. This model helps to calculate the scores of the neighboring junctions.

## 4. Proposed IJS Routing Protocol

### 4.1. System Model

In this model, we have assumed the city as a graph *G* which consists of junctions as vertices and edges as roads which connect the junctions. Vehicle and RSU beacon at a particular interval of time by which every vehicle knows about its neighboring vehicles location information. Vehicles use GPS services and maps to know their own position and roads ahead. A helping vehicle (HV) is set at every junction *J* to store the information of the vehicles passing through the junction. Vehicles forward the data to the vehicle which is in the forward position and nearer to the destination. Vehicles transmit the data to the vehicles moving in the same direction. The position of *D* is known to the source vehicle *V*_*S*_ and it is updated at every junction by the use of location services [24, 25].

### 4.2. Functionality of IJS Protocol

In the city areas, the density may be high, but what is the chance that the vehicles are connected to each other (there may be network gaps)? So, IJS is proposed to enhance the network performance by predicting link connectivity problems at an early stage. For this, a partial centralized architecture is designed in which an HV is set at every junction. HV knows about the traffic conditions ahead (between itself and neighboring junctions). As HV is a fixed vehicle it beacons and receives the information from its neighboring vehicles. HV is a trusted vehicle which is mainly fixed to receive the data from the vehicles and send the data in a selected path. When a vehicle with a data reaches the junction, it hands over the data to HV. Then, HV forwards the data to the junction which has minimum Score. This process continues until *D* is reached. These data are updated regularly with the beaconing service.

The functionality of IJS protocol starts with sending the data from the source vehicle *V*_*S*_ to the destination vehicle *V*_*D*_. *V*_*S*_ initializes the communication and calculates the SP to *V*_*D*_ using Dijkstra algorithm and forwards the data in the direction of this path. But *V*_*S*_ has only one neighboring junction which consists of an HV. Then, *V*_*S*_ forwards the data to HV through many optimal vehicles *V*_optimal_ (*V*_optimal_ is the vehicle in the range which is nearer to the neighboring junction).

After receiving the data packet, HV at the junction assumes the junction as *J*_current_ and generates a Score for every neighboring junction (*J*_neighbor_1__, *J*_neighbor_2__,…, *J*_neighbor_*j*__) (from Figure 3). HV uses the traffic information to select a new route to forward the data. As HV is aware of the vehicle identification, location, speed, and direction, HV calculates the score for every neighboring junction. HV uses EDTDM, LCM, and VPM models to generate a score. Firstly, HV uses the VPM model to find the positions of the vehicles between the two junctions. Then, HV finds a logical route of reference Route_reference_ to forward the data from one HV (HV at *J*_current_) to another HV (HV at *J*_neighbor_) by using the positions and range of the vehicles.

According to Figure 3, HV can find a *V*_optimal_ to send the data in its range. If *V*_optimal_ is searched, then it is assumed that a link *l* can be established for data transmission to *V*_optimal_. In Figure 3, *V*_optimal_ is the vehicle *V*_3_ and a link *l*_4_ can be established for data transmission for *t*_available_ time (from (8), (9), and (10)). So, if data is transmitted, it takes *t*_expected(HV,*V*_3_)_ time (from (1)). The route of reference ${\text{Route}}_{\text{reference}}=\text{HV}\overset{l_{4}}{\to }V_{3}$. So, IJS considers the link connectivity status LCS = 1 for the two vehicles, HV and *V*_3_. After *V*_3_ receives the data logically, HV continues the above steps and it finds *V*_4_ as *V*_optimal_ for vehicle *V*_3_. This signifies that a link *l*_5_ for *t*_available_ time can be established for data transmission and if data is transmitted, it takes *t*_expected(*V*_3_,*V*_4_)_ time. Now, the new route of reference is ${\text{Route}}_{\text{reference}}=\text{HV}\overset{l_{4}}{\to }V_{3}\overset{l_{5}}{\to }V_{4}$. Then, IJS sets the LCS value to 1 for vehicles *V*_3_ and *V*_4_. According to Figure 3, this process continues by applying Algorithm 1 and finally the data is logically forwarded to *J*_neighbor_ (HV) on the route $\text{HV}\overset{l_{4}}{\to }V_{3}\overset{l_{5}}{\to }V_{4}\overset{l_{6}}{\to }\text{RSU}\overset{l_{7}}{\to }V_{5}\overset{l_{8}}{\to }V_{6}\overset{l_{9}}{\to }\text{HV}$ with links *l*_4_, *l*_5_, *l*_6_, *l*_7_, *l*_8_, and *l*_9_. After the Route_reference_ is generated, HV finds the SP_(*J*_current_,*V*_*D*_)_ from the current junction *J*_current_ to *V*_*D*_ and it finds the SP_(*J*_neighbor_,*V*_*D*_)_ from neighbor junction *J*_neighbor_ to *V*_*D*_. After getting all the information, IJS calculates the score of the neighboring junction as follows:

(18) Scorej=SP(Jneighbor,VD)SP(Jcurrent,VD)+∑texpected(Jcurrent,Jneighbor)+∑LCSTotal  number  of  links,

where *j* is the number of neighboring junctions, ∑*t*_expected(*J*_current_,*J*_neighbor_)_ is the total time delay to transfer the data from HV of *J*_current_ to HV of *J*_neighbor_, and ∑LCS is the sum of all the LCS values predicted. IJS mainly considers the propagation delay and transmission delay in *t*_expected_ to calculate the score. The score is generated for every neighboring junction, except the junction through which the data has already transferred. The junction with the minimum score is selected as the next optimal junction. The optimal junction *J*_optimal_ is selected as

(19) $$\begin{array}{c} J_{\text{optimal}}=\min\left(\text{Scor}{\text{e}}_{1},\text{Scor}{\text{e}}_{2},\ldots ,\text{Scor}{\text{e}}_{j}\right). \end{array}$$

According to Figure 3, HV selects the straight path with the minimum score because the left and right paths have huge network gaps with less density of vehicles which increases the value of the score.

**Figure 2:**
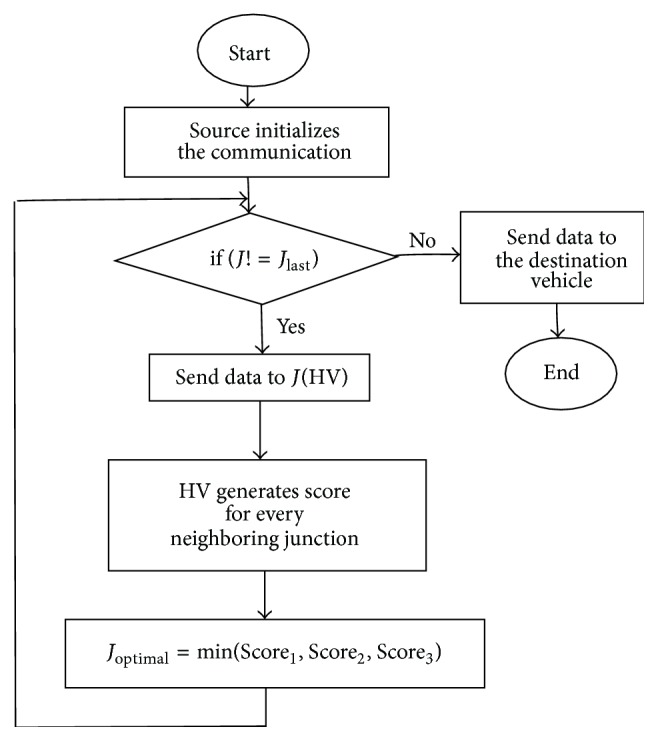
Flowchart of IJS routing protocol.

**Figure 3:**
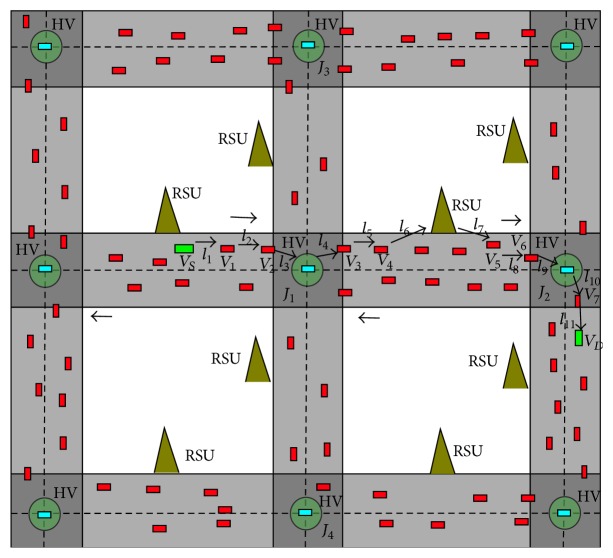
Data forwarding from *V*_*S*_ to *V*_*D*_ using IJS protocol.

Then, the data is transmitted in the direction of *J*_optimal_ by using the Route_reference_ information which is forwarded in the packet. This process continues until the last junction *J*_last_ (junction which is nearer to *V*_*D*_) is reached. From Figure 3, after data packet is delivered to HV, it forwards the data directly to *V*_*D*_ by selecting optimal vehicles.  Figure 2 shows the flowchart of IJS routing protocol. This method shows better performance by predicting the optimal junctions on the route.

IJS shows better performance by reducing the time delay to forward the data to the destination. In the city areas, due to high mobility of vehicles, there may be network gap situations in any direction. This leads to less densified region generation which disrupts the link between the vehicles. To overcome this situation, IJS uses V2I, I2V/V2I, and I2I communication. If any of these communications fails, then vehicle/infrastructure carries the data until a new vehicle is encountered in its range (Algorithm 2).

## 5. Analysis of IJS protocol

IJS protocol is analyzed by considering the junctions as vertices and edges as the roads connecting the two junctions. Then, the city is represented as a graph *G*. We consider path length, delay, and network gap to evaluate the performance of the protocol. The performance is analyzed as follows.

**Lemma 1 .**

The *SP* selected from *S* to *D* is the sum of all the intermediate distances.

**Proof**

Let the total distance calculated from *S* (source vehicle) to *D* (destination vehicle) be *d*_total_ and suppose that *S* sends data to *D* through the intermediate optimal junctions {*J*_1_, *J*_2_,…, *J*_*j*_}, where *j* = {1,2,…, *j*}, and then path *P* is represented as

(20) $$\begin{array}{c} P=S⟶J_{1}⟶J_{2}⟶\cdots ⟶J_{j}⟶D. \end{array}$$

From (20),

(21) dtotal=SJ1+J1J2+⋯+JjD=d1+d2+⋯+dj=∑j=1jdj,

where *J*_*j*_ is the last junction nearer to destination *D*.

**Lemma 2 .**

Path length increases with the increase in the number of junctions in the path.

**Proof**

Let the shortest path *P*_1_ have *j*_*P*_1__ number of junctions and let the shortest path *P*_2_ have *j*_*P*_2__ number of junctions, where *j*_*P*_1__ > *j*_*P*_2__. From (20) and (21), *P*_1_ = *S* → *J*_1_ → *J*_2_ → ⋯→*J*_*j*_*P*_1___ → *D* and *P*_2_ = *S* → *J*_1_ → *J*_2_ → ⋯→*J*_*j*_*P*_2___ → *D* and *d*_total_ for *P*_1_ and *P*_2_ is presented as

(22) dtotal1=SJ1+J1J2+J2J3+⋯+JjP1D=d1+d2+⋯+dj=∑j=1jdj,dtotal2=SJ1+J1J2+J2J3+⋯+JjP2D=d1+d2+⋯+dj=∑j=1jdj.

Let *t*_total_1__ and *t*_total_2__ be the time delays in paths *P*_1_ and *P*_2_, respectively, to send the data from *S* to *D* and it is presented as

(23) ttotal1=texpected(S,J1)+texpected(J1,J2)+⋯+texpected(JjP1,D)=t1+t2+⋯+tj=∑j=1jtj,ttotal2=texpected(S,J1)+texpected(J1,J2)+⋯+texpected(JjP2,D)=t1+t2+⋯+tj=∑j=1jtj.

As *j*_*P*_1__ > *j*_*P*_2__, from (23), *t*_total_1__ > *t*_total_2__ and hence if the number of hops (junctions) increases, distance increases.

**Lemma 3 .**

Delay increases with the increase in the number of junctions in the path.

**Proof**

From Lemma 2, as the number of junctions in the path increases, delay increases. Equation (23) shows the delays in the paths *P*_1_ and *P*_2_, respectively. It is concluded that if the junctions are not selected intelligently then the number of junctions increases and *t*_total_1__ > *t*_total_2__.

**Lemma 4 .**

Delay increases with the generation of network gaps.

**Proof**

Let the data transmission path from one junction *J*_1_ to the other be *P*_1_ = *J*_1_ → *V*_1_ → *V*_2_ → ⋯→*J*_2_, where vehicles (*V*_1_, *V*_2_,…, *V*_*n*_) denote the optimal vehicles. Path *P*_1_ has a higher density of vehicles and they are mostly connected to each other. Similarly, the data transmission path for *P*_2_ is *P*_2_ = *J*_3_ → *V*_1_ → *V*_2_ → ⋯→*J*_4_. Let the distance from *J*_1_ to *J*_2_ be the same as *J*_3_ to *J*_4_. According to the protocol, if network gaps are predicted earlier, then the delay decreases. Let *P*_1_ have *C*_*P*_1__ number of carry and forward mechanisms and let *P*_2_ have *C*_*P*_2__ number of carry and forward mechanisms, where *C*_*P*_2__ > *C*_*P*_1__.

According to the protocol, if vehicle encounters a network gap, it carries the data until a new vehicle is encountered in the path. If the number of carry and forward mechanisms increases, delay increases. So, the delay for paths *P*_1_ and *P*_2_ is represented as

(24) texpected(J1,J2)=texpected(J1,V1)+texpected(V1,V2)+tC+texpected(V2,V3)︷carry  and  forward⋯+texpected(Vn,J2),texpected(J3,J4)=texpected(J3,V1)+texpected(V1,V2)+tC+texpected(V2,V3)︷carry  and  forward+tC+texpected(V3,V4)︷carry  and  forward⋯+texpected(Vn,J4),

where *t*_*C*_ denotes the carry and forward mechanism. As path *P*_1_ has less number of carry and forward mechanisms, *t*_expected(*J*_3_,*J*_4_)_ > *t*_expected(*J*_1_,*J*_2_)_. So, the existence of network gap increases the delay.

**Lemma 5 .**

*Score* calculation uses the best parameters to select an optimal junction.

**Proof**

To calculate the Score in IJS protocol, we use the shortest path SP_(*J*_current_,*V*_*D*_)_ from the current junction *J*_current_ to *V*_*D*_ and the shortest path SP_(*J*_neighbor_,*V*_*D*_)_ from neighbor junction *J*_neighbor_ to *V*_*D*_. Paths are calculated to find the minimum distance from the junctions to the destination vehicle *V*_*D*_ to send the data in a quicker path. SP_(*J*_neighbor_,*V*_*D*_)_/SP_(*J*_current_,*V*_*D*_)_ provides the closeness of destination to the neighbor junction. Then, IJS uses *t*_expected(*J*_current_,*J*_neighbor_)_ which provides the minimum time to send a data packet from one junction to the other. This value should be the minimum. Connectivity of the vehicles is assumed by the link connectivity status LCS which shows whether the vehicles are connected or not. (∑LCS/Total  number  of  links) finds the average of the links between the vehicles.

By combining the three parameters, we find the formula for the Score in (25) and the junction with the minimum value is selected as the optimal junction. The Score for a junction is calculated as follows:

(25) Scorej=SP(Jneighbor,VD)SP(Jcurrent,VD)+∑texpected(Jcurrent,Jneighbor)+∑LCSTotal  number  of  links.

**Theorem 6 .**

The junction with the minimum *Score* is the best junction through which the data is forwarded.

**Proof**

Let a junction *J*_1_ have three neighboring junctions *J*_2_, *J*_3_, and *J*_4_. We assume that the density of the vehicles between *J*_1_ and the three neighboring junctions is *λ*_1_, *λ*_2_, and *λ*_3_, respectively. According to Lemma 5, Score of a junction depends on the connectivity of the vehicles. If in between the junctions the vehicles are connected consecutively, then it is the best path to transmit the data. For junctions *J*_2_, *J*_3_, and *J*_4_, the probability of connectivity is shown as follows:

(26) $$\begin{array}{c} \mathrm{Pr}\left(J_{1}J_{2}\right)={\left[e^{-\lambda R}\right]}^{p}\cdot {\left[1-e^{-\lambda R}\right]}^{M-1-p},\\ \mathrm{Pr}\left(J_{1}J_{3}\right)={\left[e^{-\lambda R}\right]}^{q}\cdot {\left[1-e^{-\lambda R}\right]}^{N-1-q},\\ \mathrm{Pr}\left(J_{1}J_{4}\right)={\left[e^{-\lambda R}\right]}^{u}\cdot {\left[1-e^{-\lambda R}\right]}^{Q-1-u}, \end{array}$$

where *M*, *N*, and *Q* are the number of consecutive vehicles which can be connected in between the junctions. *p*, *q*, and *u* are the number of network gaps or link failures between the junctions. We assumed that *p* > *q* > *u* and *λ*_3_ > *λ*_2_ > *λ*_1_, so we conclude that *Pr*⁡(*J*_1_ *J*_4_) has a higher value than *Pr*⁡(*J*_1_ *J*_2_) and *Pr*⁡(*J*_1_ *J*_3_)(*Pr*⁡(*J*_1_ *J*_4_) > *Pr*⁡(*J*_1_ *J*_3_) > *Pr*⁡(*J*_1_ *J*_2_)). From Lemma 4, it is proved that if there is less number of network gaps and the road is highly connected, then delay reduces.

From Lemma 5, dividing the shortest paths provides the closeness information of the destination to the source. This minimum value helps in finding the minimum Score junction. This combination of the parameters justifies the finding of the best junction through which the data is quickly transmitted.

**Theorem 7 .**

If network gaps are predicted earlier, then the delay to send the data from *S* to *D* reduces.

**Proof**

After receiving the data, let junction *J*_1_ use path *P*_1_ to send the data to *D* using the optimal junctions. Let path *P*_1_ be represented as *P*_1_ = *J*_1_ → *J*_2_ → *J*_3_ → ⋯→*J*_*j*_ → *D*, where (*J*_2_, *J*_3_,…, *J*_*j*_) are the optimal junctions. Then, the total distance is represented as

(27) $$\begin{array}{c} d_{\text{total}}=J_{1}J_{2}+J_{2}J_{3}+\cdots +J_{j}D \end{array}$$

and, according to Lemma 4, the expected delay to send the data from one junction to the other is represented by *t*_expected(*J*_current_,*J*_neighbor_)_. So, the total delay *t*_total_ for the path is represented as

(28) ttotal=texpected(J1,J2)+texpected(J2,J3)+⋯+texpected(Jj,D)=[texpected(J1,V1)+texpected(V1,V2)  +tC+texpected(V2,V3)︷carry  and  forward+tC+texpected(V3,V4)︷carry  and  forward⋯  +texpected(Vn,J2)] +[texpected(J2,V5)+texpected(V5,V6)   +tC+texpected(V6,V7)︷carry  and  forward+tC+texpected(V7,V8)︷⋯carry  and  forward   +texpected(Vn,J3)]+⋯ +[texpected(Jj,V9)+texpected(V9,V10)   +tC+texpected(V10,V11)︷carry  and  forward+tC+texpected(V11,V12)︷carry  and  forward⋯   +texpected(Vn,D)].

According to Lemma 4, if the path has less number of network gaps or link failures, then delay reduces. From (28), if the network gaps between the junctions increase, the delay increases. So, IJS selects the best junctions in the path by predicting the network gaps between the junctions earlier and selects the neighboring junction where the vehicles are mostly connected.

## 6. Simulation and Results

IJS routing protocol is simulated and compared with GyTAR, A-STAR, and GSR routing protocols. To check the performance of IJS, we have considered three performance metrics through which the performance of the protocols is evaluated. The performance metrics are discussed as follows:

1. average end-to-end delay: this is the average delay to send a data packet from the source to the destination;
2. network gap encounter: this represents the total number of network gaps encountered in a route when a data packet is sent from the source to the destination;
3. number of hops: this represents the total number of hops or vehicles used for data transmission between the source and the destination.

In this simulation, the parameters are set according to Table 1. We have considered 36 junctions and the distance between the junctions is set to be 2500 m and 3000 m. To identify the performance of the protocols, the distance between the junctions is varied. The maximum number of vehicles between the junctions is varied to evaluate the performance of the network with different densities. The speed of the vehicles is set to be 70–90 Km/H. Vehicle range is set to be 250 m and as RSU has a higher range, it is set to be 500 m. RSU is set at the center of the road. The packet size is set to be 512 bytes. The protocols are implemented using MATLAB version R2012a (7.14.0.739). The protocols are implemented in the perfect conditions and the performance is evaluated according to EDTDM, LCM, and VPM models discussed above.

In this simulation environment, we have assumed the ideal conditions in which link is established with no disturbance when an optimal vehicle is encountered in the range. We considered perfect conditions where no dropping of packets and contention occurs in the network.

**Figure 4:**
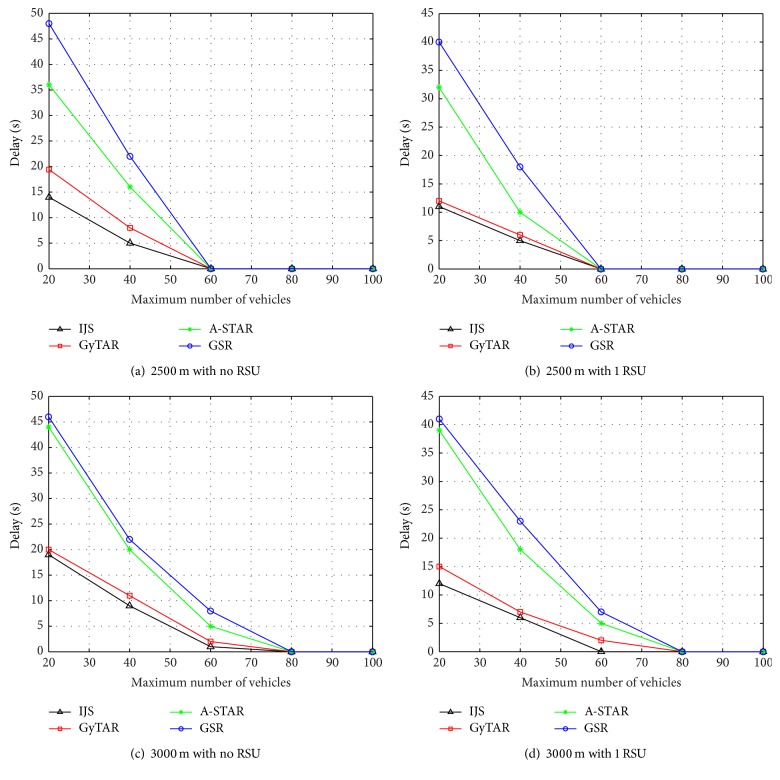
Simulation results for average end-to-end delay in IJS, GyTAR, A-STAR, and GSR.

In these scenarios, we have set the distance between the junctions to be 2500 m and 3000 m by varying the number of RSUs with no RSU and 1 RSU. According to Figures 4(a), 4(b), 4(c) and 4(d) IJS shows a less delay than GyTAR, A-STAR, and GSR routing protocols. In this, we identify that when the density of vehicles increases, the delay reduces due to high chance of link establishment. When the maximum density between the vehicles is set to 60, 80, and 100, the delays of the protocols varied by a less difference.  Figure 4(b) shows a less delay than Figure 4(a) because RSU reduces the number of hops. As the distance between the junctions increases, the delay increases due to less density of vehicles in the area. From Figures 4(a) and 4(c) we conclude that Figure 4(c) has a higher delay than Figure 4(a).

**Figure 5:**
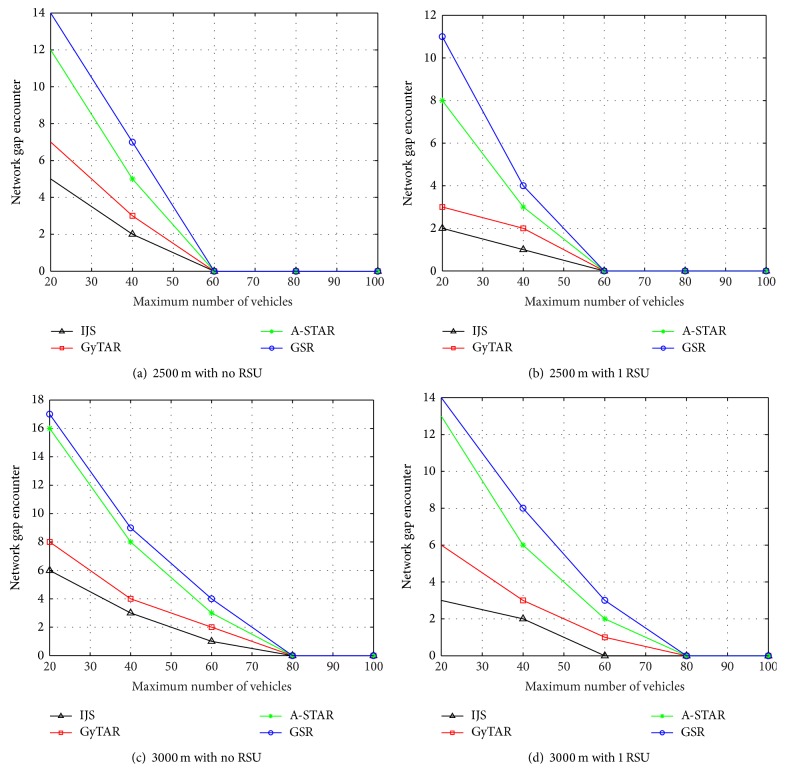
Simulation results for network gap encounter in IJS, GyTAR, A-STAR, and GSR.

In Figures 5(a), 5(b), 5(c) and 5(d) IJS encounters less number of network gaps. When the vehicle population increases, the network gap reduces because of the high chance of encountering a vehicle in the range. This concludes that IJS detects the network gaps at an early stage and protects the network from link disruption problems. The probability of connectivity *Pr*⁡ increases with the decreases in the number of network gaps. The average number of network gaps in Figure 5(a) for IJS, GyTAR, A-STAR, and GSR is 1.4, 2, 3.4, and 4.2, respectively, and, for Figure 5(b), the average number of network gaps is 0.6, 1, 2.2, and 3, respectively. As RSU is set between the junctions, network gap reduces. This signifies that RSU helps in reducing the network gaps. Figure 5(a) shows a less number of average network gaps than Figure 5(c) which has an average of 2, 2.8, 5.4, and 6 for IJS, GyTAR, A-STAR, and GSR routing protocols, respectively.

**Figure 6:**
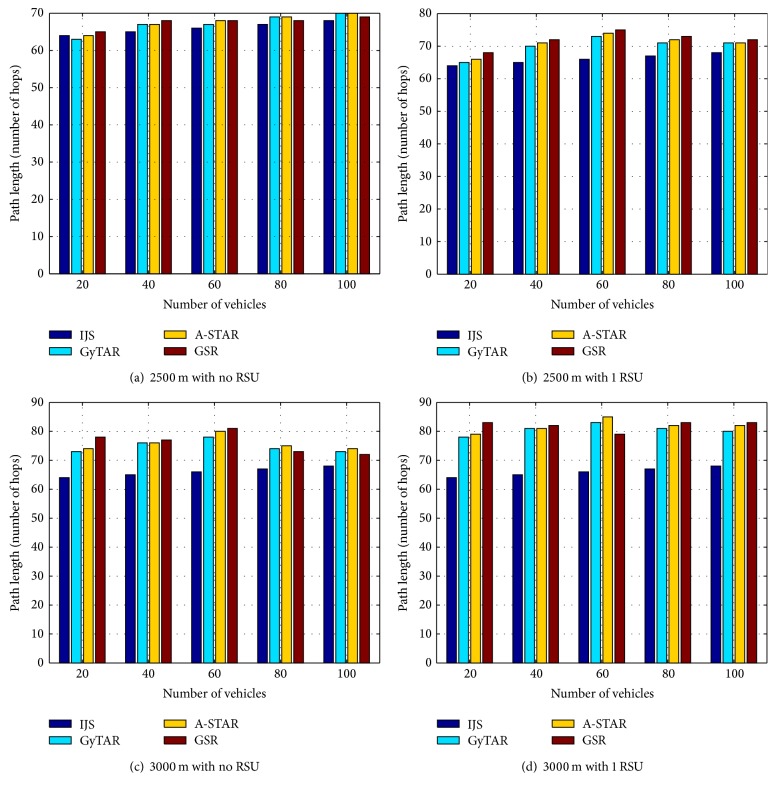
Simulation results for number of hops in IJS, GyTAR, A-STAR, and GSR.

In Figures 6(a), 6(b), 6(c) and 6(d) IJS has less number of hops than GyTAR, A-STAR, and GSR routing protocols. As vehicles density increases, the number of hops reduces due to high chance of availability of vehicles. This helps in selecting the optimal vehicles in the range.  Figure 6(a) shows more number of hops than Figure 6(b) because the presence of RSU reduces the number of hop counts.  Figure 6(a) shows less number of hops than Figure 6(c) because as the distance between the junctions increases, the chance of getting vehicles in the range reduces.

## 7. Conclusion

IJS is an intelligent routing protocol for VANET, in which HV predicts the most connected route to the destination with less delay. This protocol will be a better routing protocol for vehicular communication providing ITS services (e.g., accident warning services) to the passengers and drivers. IJS detects the network gap at an early stage before sending the data packet. By considering the population between the junctions and finding a logical Route_reference_, the score for every neighboring junction is calculated and the minimum score junction is selected as the next junction through which the data is forwarded. This strategy helps the vehicles to send their data in a quicker path. This reduces the end-to-end delay, network gap encounter, and number of hops. Simulation results show that IJS performance is better than GyTAR, A-STAR, and GSR routing protocols. This protocol promises a better solution to provide services to the drivers and passengers.

## Figures and Tables

**Algorithm 1:**
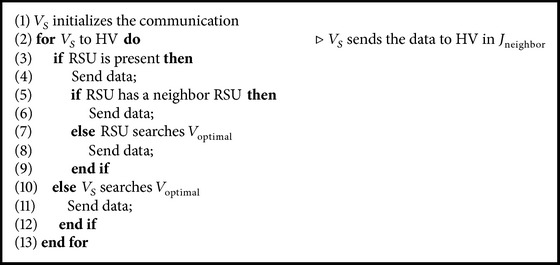
Data forwarding from *V*_*S*_ to HV.

**Algorithm 2:**
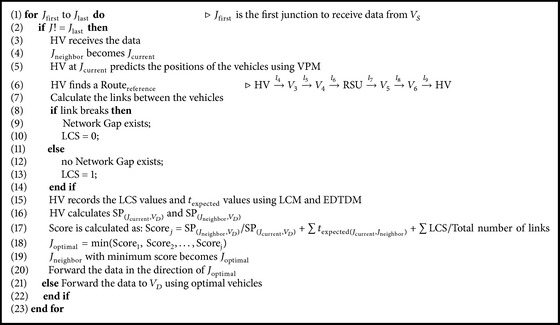
Score generation.

**Table 1:** Simulation environment.

Parameter	Parameter value
Number of junctions	36
Distance between the junctions	3000 m
Number of vehicles between the junctions	20–100
Vehicle speed	70–90 Km/H
Vehicle range	250 m
Number of RSUs	1
RSU range	500 m
Packet size	512 bytes

## Notations

*V*: Vehicle
*J*: Junction
*S*: Source
*D*: Destination
SP: Shortest path
HV: Helping vehicle
*R*: Range
*λ*: Density
Score: Score
*t*_expected_: Expected delay
*t*_*C*_: Carrying time
LCS: Link connectivity status
Route_reference_: Logical route reference
*P*: Path
*J*_current_: Current junction
*J*_neighbor_: Neighbor junction
*J*_optimal_: Optimal junction
*V*_optimal_: Optimal vehicle
*d*: Distance
*v*: Velocity
Pr: Probability of connectivity
*L*: Message length
*r*: Channel rate.
